# Widespread pyrethroid resistance in *Varroa destructor* in Türkiye: a molecular warning

**DOI:** 10.1007/s10493-026-01132-z

**Published:** 2026-04-01

**Authors:** Sezer Yalcin, Taylan Dogaroglu, Evin Gunenc, Ersin Dogac

**Affiliations:** 1 Institute of Science, Department of Molecular Biology and Genetics, Mugla Sıtkı Koçman University, Mugla, Turkey; 2 Department of Plant and Animal Production, Beekeeping Program, Ula Ali Koçman Vocational School, Muğla Sıtkı Koçman University, Mugla, Turkey; 3 Faculty of Science, Department of Molecular Biology and Genetics, Mugla Sıtkı Koçman University, Mugla, Turkey

**Keywords:** *Varroa destructor*, Pyrethroid resistance, PCR-RFLP, *Vgsc*, Türkiye

## Abstract

This study examined the prevalence of pyrethroid resistance mutations in *Varroa destructor* (Anderson and Trueman 2000) populations across ten Turkish provinces, utilizing PCR-RFLP assay. By targeting codon 925 in the voltage-gated sodium channel (*vgsc*) gene, a recognized site for knockdown resistance (*kdr*), we identified a notably high frequency of resistant alleles within the field populations. Among the 800 mites analyzed, 83% were homozygous resistant, 14% were heterozygous, and only 3% were susceptible. Regional analysis indicated the highest resistance levels in Eastern Anatolia (94%), followed by Central Anatolia (80%) and Marmara (71%). The widespread fixation of resistance alleles is likely attributable to the excessive and repeated use of flumethrin-treated strips, which exert strong selection pressure. The low heterozygosity rate, observed at only 14%, supports the hypothesis of directional selection and potential genetic bottlenecks in these populations. These findings are consistent with patterns reported in other Mediterranean countries, indicating a broader regional trend. Overall, this study presents comprehensive and concerning data on the resistance of Turkish *Varroa* populations, reinforcing global calls for sustainable and adaptive mite management strategies and providing a valuable reference for policymakers and beekeepers.

**Supplementary Information:**

The online version contains supplementary material available at 10.1007/s10493-026-01132-z.

## Introduction

*Varroa destructor* (Anderson and Trueman 2000) is a cosmopolitan parasitic mite that causes significant brood and adult mortality, bee malformations (Marcangeli et al. 1992), colony weakening, and reduced worker bee lifespan in *A. mellifera* colonies (Keszthelyi et al. 2021). These mites represent one of the most formidable challenges in the beekeeping industry today; climate change has shortened natural broodless periods, while the increasing prevalence of bee viruses has further complicated control efforts. As devastating parasites, they feed on both adult and developing bees, transmitting several harmful viruses including Deformed Wing Virus (DWV), Acute Bee Paralysis Virus (ABPV), Israeli Acute Paralysis Virus (IAPV), Kashmir Bee Virus (KBV), Black Queen Cell Virus (BQCV), and Sacbrood Virus (SBV), with DWV being the most widespread and damaging (Locke et al. 2021; Robi et al. 2023).

Due to these detrimental effects, beekeepers worldwide have struggled with sustainable *Varroa* management for decades. While miticides were highly effective in the past, their efficacy has diminished due to the development of resistance (Higes et al. 2020; Benito-Murcia et al. 2022). To counteract these infestations, beekeepers rely on miticides that target specific molecular pathways in the parasite (Calatayud-Vernich et al. 2018). Miticides target mites through various molecular mechanisms. By disrupting these essential components of the central nervous system, these contact-based agents—disseminated through the colony by the bees from strips—induce complete paralysis and subsequent death (Bahreini et al. 2025).

However, the intensive and prolonged use of these chemical treatments has led to a significant global increase in resistant *Varroa* populations. Resistance has been documented against organophosphates, amitraz, and pyrethroids (González-Cabrera et al. 2013, 2016, 2018; Hubert et al. 2014; Alissandris et al. 2017; Jack and Ellis 2021; Hernández-Rodríguez et al. 2021; Millan-Leiva et al. 2021; Vlogiannitis et al. 2021; Almecija et al. 2022; Hernandez- Rodríguez et al., 2022; Marsky et al. 2024; Bahreini et al. 2025; Celikkol and Dogac 2025).

Pyrethroid resistance, specifically *kdr*-*type*, is primarily linked to mutations at codon 925 of the *vgsc* gene. To date, three specific resistance-associated alleles—L925V, L925I, and L925M—have been thoroughly characterized (González-Cabrera et al. 2013, 2016). The L925V mutation has been identified in several regions across continental Europe, including Southern and Central England (González-Cabrera et al. 2013), Czechia (Hubert et al. 2014; Stara et al. 2019), Greece (Alissandrakis et al. 2017), Italy (Panini et al. 2019), Belgium (Vlogiannitis et al. 2021) and Turkiye (Koc et al. 2021). The L925I variant has been reported in the United States (González-Cabrera et al. 2013), Greece (Alissandrakis et al. 2017)d rkiye (Erdem et al. 2024; Koç et al. 2021). In contrast, the L925M mutation has only been observed in the United States (González-Cabrera et al. 2013), Japan (Ogihara et al. 2021)d rkiye (Erdem et al. 2024). An additional amino acid substitution, M918L, has been reported in Spain, alongside L925V (Benito-Murcia et al. 2022). Moreover, other potential mutations, namely I1752V, F1528L, M1823I, and L1596P, have been detected in resistant mites from Michigan and Florida in the United States; however, their functional relevance to resistance has not yet been conclusively established (Dong et al. 2014; Rinkevich 2020; Wang et al. 2003).

The L925V mutation has been reported across numerous regions worldwide and is considered a major molecular marker for target-site pyrethroid resistance (González-Cabrera et al. 2018; Hubert et al. 2014). Although earlier studies from specific parts of Türkiye have documented the presence of resistant *Varroa* genotypes (Koç et al. 2021; Yarsan et al. 2024; Celikkol and Dogac 2025), comprehensive large-scale national data remain limited. Furthermore, recent findings suggest that insect populations often undergo genetic bottlenecks due to restricted gene flow and strong selection imposed by long-term acaricide use, accelerating the fixation of resistance alleles (Lin et al. 2025). In addition to selection pressure from chemical treatments, *Varroa* populations interact with various biotic and abiotic stressors, which influence host–parasite dynamics, mite survival, and the maintenance of resistant alleles (Lin et al. 2024). Recent evidence further indicates that exposure to natural compounds such as essential oils can trigger substantial changes in mite gene expression—including genes associated with detoxification and stress responses—highlighting the complexity of *Varroa* resistance biology (Shen et al. 2025).

Given the continued reliance on flumethrin-based control in Türkiye and the absence of up-to-date nationwide resistance surveillance, evaluating the current distribution and frequency of resistance alleles is essential. Therefore, this study investigates the prevalence of the L925V mutation in *Varroa* populations collected from ten provinces representing major beekeeping regions of Türkiye. The objectives of this research were to: (i) determine genotype and allele frequencies of the L925V mutation at a national scale, (ii) assess regional differences in resistance distribution, and (iii) provide evidence-based insights to support sustainable *Varroa* management strategies.

## Materials and methods

### Sampling areas and colony selection

**Fig. 1:**
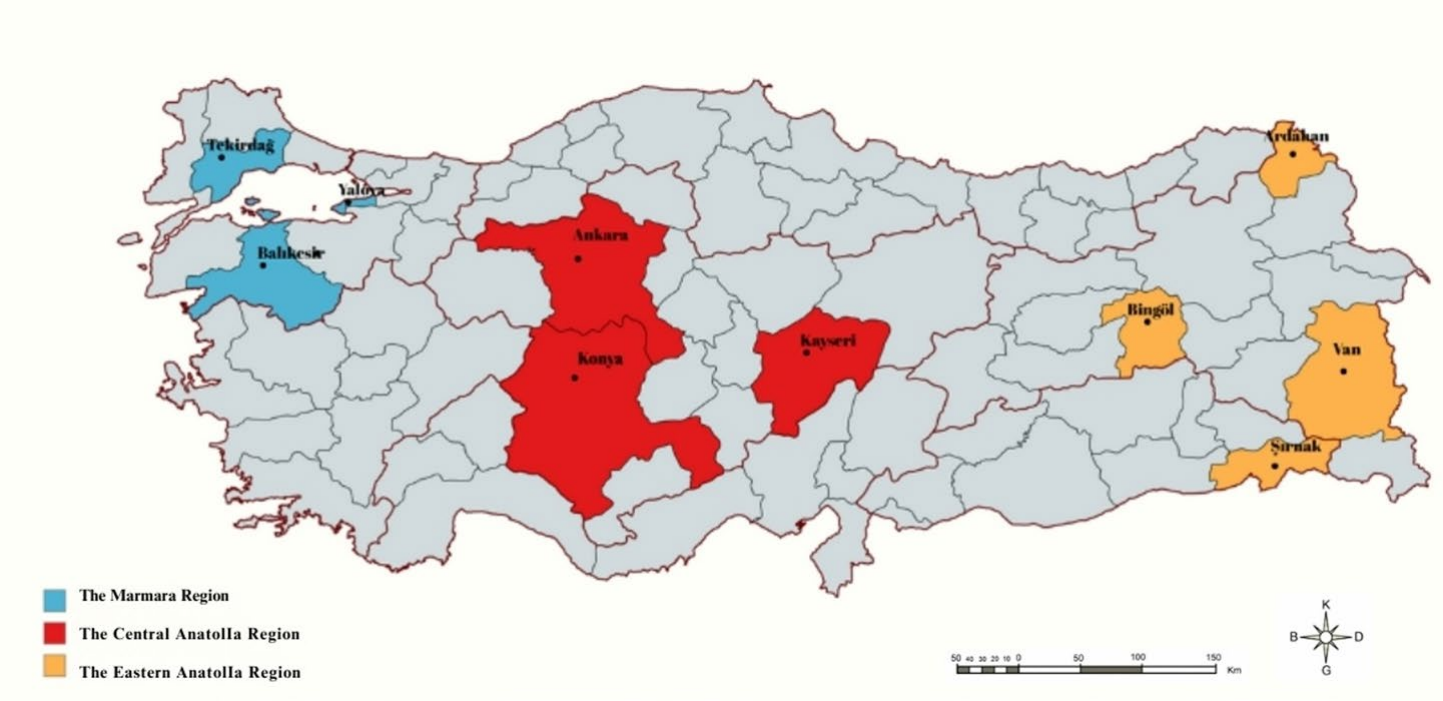
Collection sites of *Varroa* samples. (Marmara region; blue, Central Anatolia region; red and Eastern Anatolia region; yellow)

This study was conducted across ten provinces spanning three important beekeeping regions in Türkiye: Marmara (Yalova, Balıkesir, Tekirdağ), Central Anatolia (Ankara, Konya, Kayseri), and Eastern Anatolia (Ardahan, Van, Bingöl, Şırnak) (Fig. 1). We prioritized provinces from regions that had been underrepresented in our previous studies. Provinces were selected to (i) capture areas with high beekeeping activity and (ii) provide broad east–central–west coverage to assess the current status of resistance across Türkiye. In each of the ten provinces, mites were collected from a single apiary by sampling eight hives. Approximately 100 mites were initially collected per hive; from these, 10 mites per colony were randomly selected for laboratory analyses, yielding 80 mites per province (800 mites in total). To minimize mite transmission through robbing and drifting among adjacent colonies, we deliberately selected apiaries located at least 100 m away from any other managed apiaries, and within each apiary, only colonies with a minimum inter-colony distance of 5 m were included in *Varroa* sampling. This multi-colony sampling strategy was implemented to ensure a more representative assessment of the mite population within the apiary and to minimize potential bias stemming from individual hive dynamics.

### Acaricide treatment background

All sampled colonies had a documented history of flumethrin application outside the honey production season. Because pyrethroids exert strong selective pressure on *Varroa* populations, sampling colonies with known flumethrin exposure provides an ecologically meaningful assessment of resistant allele distributions (González-Cabrera et al. 2018). To minimize immediate knockdown bias and to allow colonies to stabilize after chemical exposure, sampling was deliberately conducted 2–3 weeks after the last flumethrin treatment during September-October. This timing increases the likelihood of capturing mites that naturally remained in the hive following treatment and represents a realistic field composition of resistant and susceptible individuals (Rinkevich 2020).

### Mite collection procedure

Mites were collected using the powdered sugar method, a non-destructive technique widely used for recovering phoretic, live adult mites from worker bees. To dislodge *Varroa* mites from adult bees, approximately 20–30 g of powdered sugar was evenly applied over the hive frames and allowed to act for 30 min; mites that fell onto the plastic hive bottom boards were subsequently collected. Following the powdered sugar treatment, detached *Varroa* mites were retrieved and transferred into 80% ethanol, then stored at − 20 °C until DNA extraction for preservation.

### DNA isolation, amplification and genotyping of *vgsc* fragment

A 590 bp fragment encompassing codon 925 of the *vgsc* gene was amplified using primer pairs widely employed in pyrethroid resistance diagnostics (González-Cabrera et al. 2013). The forward primer 1273f (5′-AAG CCG CCA TTG TTA CCA GA-3′) and reverse primer ECR (5′-GTG AGA AGC GCT ACA ATG AGC-3′) were used to selectively amplify the target region (Celikkol and Dogac 2025). PCR reactions were prepared in a 25 µL volume containing 1× PCR buffer, 2.0 mM MgCl₂, 0.2 mM of each dNTP, 0.4 µM of each primer, 1 U of Taq DNA polymerase, and approximately 50 ng of genomic DNA. The thermal cycling protocol consisted of an initial denaturation at 95 °C for 2 min, followed by 35 cycles of 95 °C for 30 s, 60 °C for 20 s, and 72 °C for 1 min, with a final extension step at 72 °C for 5 min. PCR products were subsequently confirmed through electrophoresis on 1% agarose gels to verify successful amplification.

### Restriction enzyme digestion and genotyping

Genotyping of the L925V mutation was performed by digesting PCR products with the restriction enzyme *SacI*, which selectively cleaves the wild-type (susceptible) allele but leaves the mutant (resistant) allele intact, enabling clear genotype differentiation (Hubert et al. 2014). Digestion reactions were conducted at 37 °C for 2 h following the manufacturer’s protocol. After digestion, samples were separated on 2% agarose gels to resolve the fragment patterns. Genotypes were then assigned based on the observed bands compared with the expected profiles for each allele.

### Assessment of gel images and statistical analyses

Allele and genotype frequencies were calculated for each province. Regional comparisons were performed using the Kruskal–Wallis test, followed by Dunn’s post hoc analysis when applicable. A significance threshold of *p* < 0.05 was used. Statistical analyses were conducted in SPSS v26 and R v4.2.

## Results

**Fig. 2:**
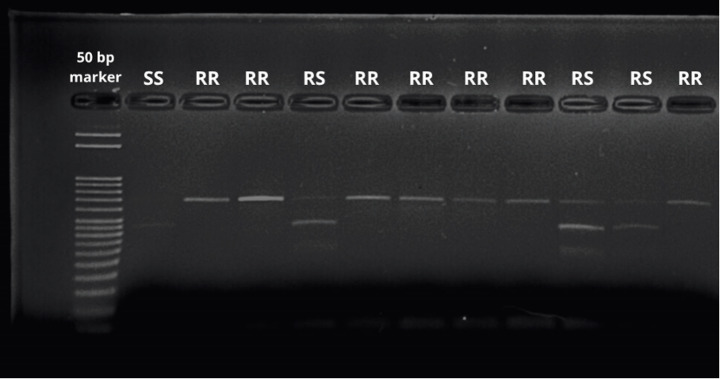
*SacI* digestion profile of Yalova province

A total of 800 mite specimens, collected from ten provinces across three regions of Türkiye, were analyzed through enzymatic digestion using *SacI*. Specimens displaying two bands at 437 and 153 bp were classified as homozygous for the susceptible allele (SS), whereas those with a single 590 bp band were identified as homozygous for the resistant allele (RR). Samples exhibiting all three bands (590, 437, and 153 bp) were considered heterozygous (RS genotype). Given that *kdr* and *super-kdr* resistance traits are recessively inherited, only individuals with a single 590 bp band were deemed resistant (Davies et al. 2007) (Fig. 2).

**Fig. 3:**
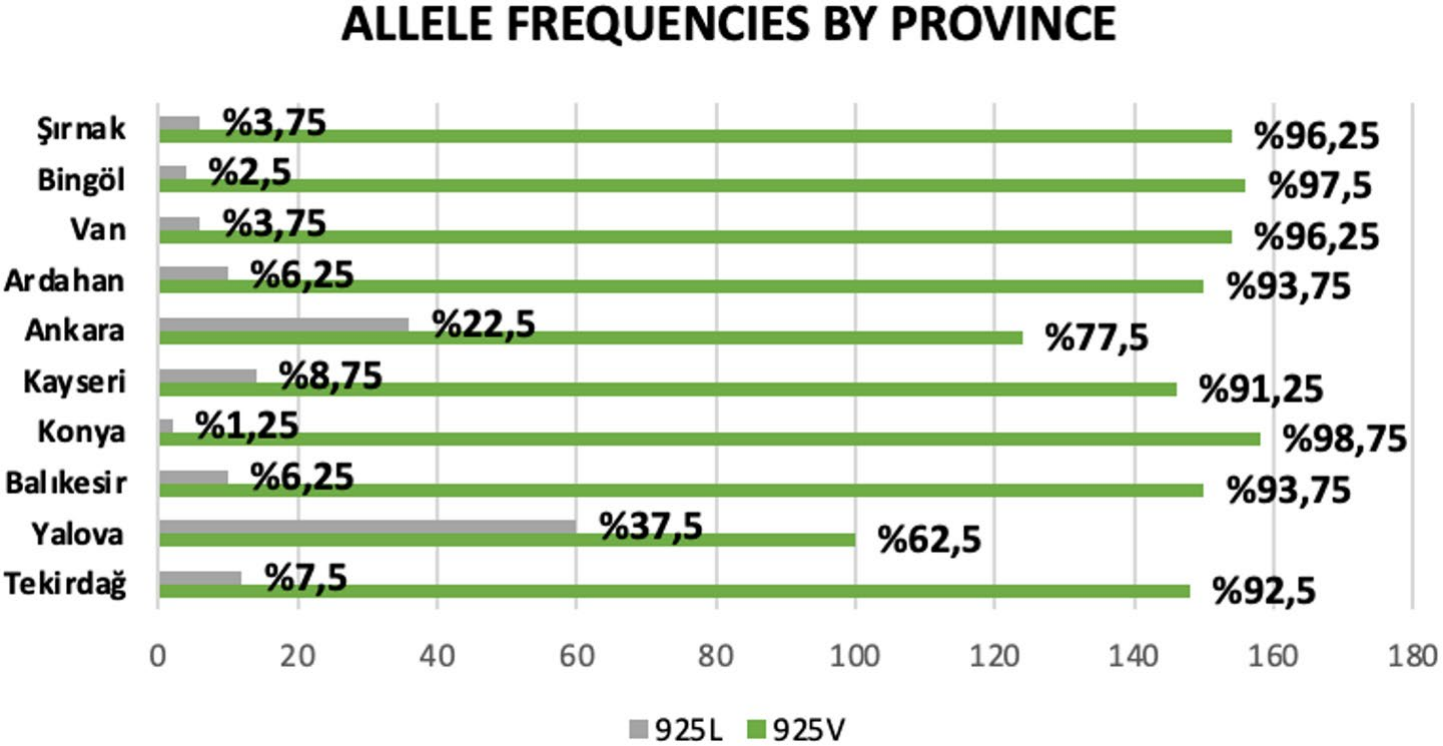
The distribution of allele numbers and frequencies is presented by province. Susceptible: gray, resistant: green

Among the 800 *Varroa* specimens examined, 662 (approximately 83%) exhibited the homozygous resistant genotype, 116 (14%) were heterozygous, carrying one susceptible allele, and only 22 (3%) were homozygous for the susceptible variant. Notably, no individuals with the homozygous susceptible genotype (SS) were identified in samples from Tekirdağ, Balıkesir, Konya, Kayseri, Van, and Bingöl (Table S1; Fig. 3). Based on allele frequency analysis, Konya exhibited the highest prevalence of the resistant allele at 98.75%, followed by Bingöl at 97.5%, and Van and Şırnak at 96.25%. In contrast, the highest frequencies of the susceptible allele were recorded in the Yalova (37.5%), Ankara (22.5%), and Kayseri (8.75%) populations (Online Resource 1; Fig. 3).

**Fig. 4:**
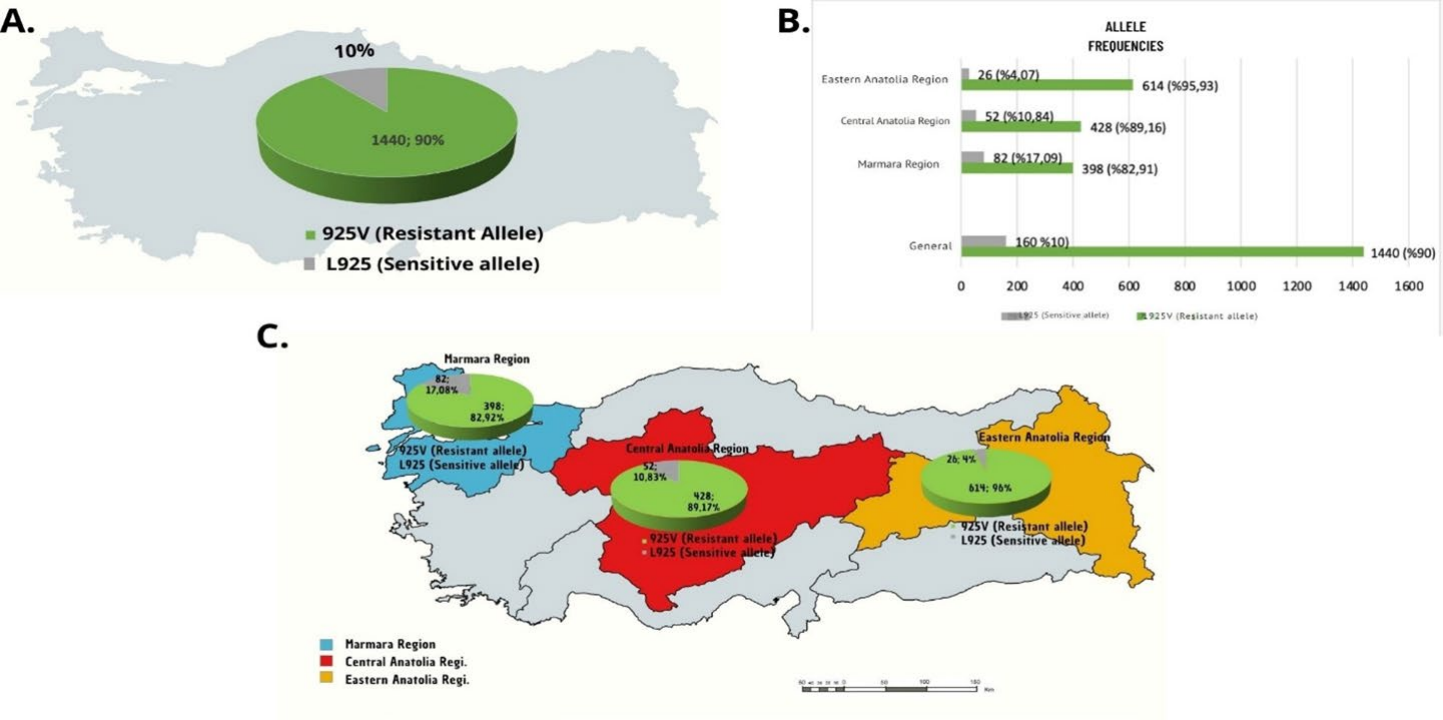
Illustrative representation of the frequencies of resistant and susceptible alleles across all sampled populations (**A**), as well as by specific regions (**B**) and (**C**)

Regional comparisons revealed that *Varroa* mites collected from the Eastern Anatolia region exhibited the highest level of resistance to pyrethroids, with 300 individuals identified as homozygous resistant and a resistant allele frequency of 95.93%. The Central Anatolia region followed with 192 homozygous resistant mites and an allele frequency of 89.16%. The Marmara region showed the lowest resistance among the three, with 170 homozygous resistant individuals and a corresponding frequency of 82.91%. Overall, the national average frequencies of resistant and susceptible alleles in Türkiye were 90% and 10%, respectively (Fig. 4).

Normality and variance homogeneity were tested using Shapiro–Wilk and Bartlett’s tests, respectively, prior to each analysis. According to the analysis results, the parametric test conditions were not met for the resistance mutation frequency data between provinces and regions. Therefore, the non-parametric Kruskal-Wallis test was used instead of the parametric tests. The analyses revealed significant differences in resistance frequency between provinces (*p* = 0.0026). The Dunn multiple comparison test was used to examine these differences in detail. According to the Dunn test results, the difference in resistance mutation frequency between Tekirdağ and Konya provinces was statistically significant (*p* = 0.0438). Except for Yalova and Ankara (*p* = 0.5824) and Tekirdağ (*p* = 0.0668), there were significant differences in resistance frequency among all other provinces. The difference in resistance mutation frequency between Ankara and Konya, Van, and Bingöl provinces was statistically significant (*p* = 0.001, 0.0145, and 0.0041, respectively). No statistically significant differences were found between the other provinces (*p* > 0.05). Table 1 presents the results of the statistical analysis of the provinces in the Marmara, Central Anatolia, and Eastern Anatolia regions.

**Table 1:** Statistical analysis revealed significant differences in the frequency of resistance mutations across provinces and regions (*p* < 0.001)

Marmara Region		Central Anatolia Region			Eastern Anatolia Region			
Provinces	Mean ± St. Dev.	Provinces	Mean ± St. Dev.		Provinces			Mean ± St. Dev.
Tekirdağ	0,925 ± 0,000	Konya	0,988 ± 0,007		Bingöl			0,975 ± 0,014
Yalova	0,625 ± 0,029	Kayseri	0,913 ± 0,051		Ardahan			0,938 ± 0,022
Balıkesir	0,938 ± 0,007	Ankara	0,775 ± 0,029		Van			0,963 ± 0,007
					Şırnak			0,938 ± 0,022
Regions	Marmara		Central Anatolia					Eastern Anatolia
	0,829 ± 0,044		0,892 ± 0,032					0,959 ± 0,009

The Kruskal-Wallis test was used for the statistical analysis of resistance mutation frequencies by region. The analysis revealed a statistically significant difference in resistance mutation frequency between regions (*p* = 0.0301). Comparisons between regions were then performed using the Dunn test. According to the Dunn multiple comparison test, the difference in resistance mutation frequency was statistically significant between the Marmara and Eastern Anatolia regions (*p* = 0.0085). The Eastern Anatolia Region showed a higher resistance mutation frequency compared to the Marmara Region (Table 1).

## Discussion

In *Varroa* destructor, a link between tau-fluvalinate resistance and *vgsc* mutations was first demonstrated by González-Cabrera et al. (2013) and Hubert et al. (2014). Specifically, the L925V substitution was found in all mites that survived pyrethroid treatment, whereas its frequency was notably lower in untreated populations. This particular L925 site is situated within a well-documented resistance “hotspot” in the proposed pyrethroid-binding region of the *vgsc* (González-Cabrera et al. 2013), underscoring its critical role in the development of pyrethroid resistance in *Varroa* populations in the field.

In this study, which examined the overall situation in Türkiye, resistance mutations were identified in all sampled populations. Among the 800 samples analyzed using the PCR-RFLP method, the frequency of resistant alleles was found to be 90%. This significant presence of resistance variants suggests that continued reliance on pyrethroid-based treatments alone could rapidly undermine the effectiveness of *Varroa* control. These findings reveal particularly high resistant-allele frequencies in Central and Eastern Anatolia, highlighting the need to reassess acaricide use.

Apiaries and individual honeybee colonies operate within an open ecological framework, where the population dynamics of *Varroa* are continually influenced by the ongoing exchange of mites between colonies. While some transmission may occur indirectly via shared environmental sites, such as flowers, the majority of mite transfer is achieved through direct interactions, particularly when honey bees inadvertently carry mites between colonies (Peck and Seeley 2019). Such transfers commonly result from behaviors such as robbing and drifting. Drifting occurs when drones or worker bees mistakenly enter a neighboring colony, leading to the unidirectional transmission of mites. In contrast, robbing, where bees invade other colonies to steal honey, enables bidirectional mite exchange (Vlogiannitis et al. 2021). Additionally, the introduction of new colonies into apiaries, often undertaken to replenish winter losses, may inadvertently introduce non-native *Varroa* genotypes into the colonies. Transporting colonies for activities such as crop pollination further increases the likelihood of contact with genetically distinct mite populations, promoting the spread and diversification of resistant strains. This constant mite exchange facilitates the rapid dispersal of resistance alleles even into apiaries with conservative chemical use.

In line with this rapid dispersal, previous research conducted in Türkiye has consistently demonstrated the prevalence of resistance mutations in the *vgsc* gene. For instance, Koç et al. (2021) identified resistance mutations in over 75% of populations across 17 locations in the provinces of Ordu, Muğla, Eskişehir, Zonguldak, and Ankara, although these studies did not specify the levels of resistance. Erdem et al. (2024) reported that 80% of 44 *Varroa* populations from 21 provinces exhibited resistance mutations. Yarsan et al. (2024) examined resistance levels in samples from seven locations where pyrethroid treatments had been applied, reporting resistance levels ranging from 51% to 94% in Ordu and Muğla provinces. Celikkol and Dogac (2025) found that resistance allele frequencies varied between 58.75% and 96.25% in *Varroa* samples from nine provinces across three regions of Türkiye. Additionally, Sorucu et al. (2025) detected target site mutations in all samples of *Varroa* individuals from Muğla province. In the present study, the frequency of the resistance allele ranged from 62.5% to 98.75% in samples collected from 10 provinces in three regions. These findings indicate significant variability in resistance levels across provinces, suggesting that current application doses may no longer be effective and that alternative control strategies are necessary. Without changes in treatment practices, particularly the continued use of flumethrin-based products, the persistence and eventual fixation of resistance alleles in *Varroa* populations is increasingly probable. However, these differences may be attributed to factors such as sample size, treatment methods, frequency of application, and climatic conditions. Consequently, it is imperative to conduct more extensive and detailed studies to accurately monitor the development of resistance. Future research should integrate long-term molecular monitoring with phenotypic bioassays, multi-drug resistance screening, regional and climatic analyses, and detoxification enzyme assessments—including transcriptomic analyses—to establish a comprehensive early-warning system for miticide resistance.

Analyses of *vgsc* genotypes across several *Varroa* populations revealed high allele frequencies of resistant variants (L925V, L925I) and a marked scarcity of heterozygous individuals, indicating low heterozygosity at this resistance locus. For example, in Spanish populations, nearly all mites carry homozygous L925V + M918L mutations with negligible heterozygosity, indicating a strong selection pressure from prolonged pyrethroid use (Benito‑Murcia et al., 2022). In Türkiye, over 80% of the sampled mite populations contained at least one resistance allele (L925V or L925I), and many sampling locations lacked any wild-type (susceptible) alleles, consistent with the fixation of resistance and little to no heterozygosity observed (Erdem et al. 2024). Furthermore, because *kdr-type* resistance is a recessive trait, only individuals homozygous for the mutant allele (RR) exhibit a resistant phenotype, while heterozygotes (RS) remain phenotypically susceptible to pyrethroids (Benito-Murcia et al. 2022). This explains why pyrethroid treatments may actively deplete heterozygous individuals from the population, further pushing resistance alleles toward fixation. The low prevalence of heterozygotes (14%) observed in our study—which aligns with the low frequencies reported by Alissandrakis et al. (2017) and Panini et al. (2019)—can be further attributed to the high levels of inbreeding and haplodiploid inheritance mechanisms characteristic of *Varroa*. From a seasonal perspective, Beaurepaire et al. (2017) noted that heterozygous genotypes tend to be more abundant in seasons with lower progeny numbers, such as during autumn or early winter. These seasonal shifts are significant for managing *Varroa* populations because pyrethroid insecticides can be applied most effectively when heterozygous mite numbers are at their peak. The increasing resistance of *Varroa* mites to these insecticides represents an escalating threat to honey bee colony survival. For instance, Bak et al., (2012) found that the failure to identify pyrethroid resistance in *Varroa* mites in time resulted in a 75% mortality rate in honey bee colonies during winter in Poland.

Regional differences in resistance allele frequencies were detected, although the overall trend remained consistent—resistance was dominant everywhere. Eastern and central provinces exhibited comparatively higher RR frequencies. In our study, we observed resistant allele frequencies of 71%, 80%, and 94% in populations from the Marmara, Central Anatolia, and Eastern Anatolia regions, respectively. The harsher climate in Eastern Anatolia leads to more intensive and frequent chemical treatments by beekeepers to prevent colony losses, which creates a stronger selection pressure compared to the more temperate Marmara region. These geographic patterns are likely influenced by a combination of acaricide exposure, apiary management practices, colony movement, and treatment synchrony across neighboring regions. Such factors collectively drive the selection and spread of resistance alleles.

Emerging evidence suggests that insect populations can undergo genetic bottlenecks in response to strong acaricide pressure, which reduces genetic diversity and accelerates allele fixation (Lin et al. 2025). The low heterozygosity and scarcity of susceptible alleles observed in this study reflect this scenario, indicating a narrowed genetic structure dominated by resistant genotypes. This outcome may reduce the potential for reversion to susceptibility, even in the absence of pyrethroid use.

In *Varroa* populations, regional differences in insecticide resistance rates are strongly influenced by climatic conditions and the use of acaricides. Studies across Italy (Panini et al. 2019)d rkiye (Celikkol and Dogac 2025) have consistently shown that resistance allele frequencies are significantly higher in southern regions than in northern areas. This pattern is primarily attributed to year-round brood production in warmer climates, which allows the *Varroa* mites to reproduce continuously. Consequently, more mite generations per year experience repeated selection pressure from acaricide exposure, particularly pyrethroids, leading to the rapid spread and fixation of resistance mutations (e.g., L925V and L925I in the *vgsc* gene). Overall, the interaction between climate, brood phenology, and treatment pressure creates distinct geographic patterns in resistance development. These findings underscore the importance of tailoring *Varroa* management strategies to local environmental and beekeeping conditions to delay the spread of resistance.

The discovery of high resistance allele frequencies across *Varroa* populations suggests that conventional pyrethroid-based treatments are becoming increasingly ineffective. Rotating acaricide classes with different modes of action can reduce the selection pressure on a single resistance pathway (Rinkevich 2020). Alternating chemical treatments with organic acids (e.g., oxalic or formic acid) or essential oils (e.g., camphor and thymol) helps slow resistance buildup, as these compounds do not select for the same mutations associated with pyrethroid resistance (Rosenkranz et al. 2010). For instance, Milani and Della Vedova (2002) conducted a study in northern Italian apiaries where pyrethroids were not used. Over a three-year period (1996–2000), they observed a substantial reduction in the proportion of resistant mites, with a tenfold decrease in resistant populations. Similarly, González-Cabrera (2018) examined the relationship between insecticide application and changes in genotypic frequency. Their study revealed that when colonies usually treated with Apistan were left untreated for eight months, the percentage of susceptible (SS) genotypes in the population increased from 34.6% to 70.8%. Similarly, Almecija et al. (2022) found that in *Varroa* populations not exposed to tau-fluvalinate for over two years, 97% of the mites exhibited susceptible genotypes, suggesting that *Varroa* mites can quickly regain susceptibility to tau-fluvalinate after cessation of treatment.

The role of biotic and abiotic stressors in shaping resistance dynamics should also be considered. Environmental stressors such as temperature fluctuations, nutritional limitations, viral infections, and host immune status can influence mite survival and reproductive success (Lin et al. 2024). These interactions may indirectly support the persistence of resistant genotypes, especially in weakened colonies that are less capable of suppressing mite populations. Furthermore, recent transcriptomic analyses have demonstrated that exposure to essential oils can induce detoxification-related gene expression in *Varroa* (Shen et al. 2025), suggesting that resistance phenotypes may involve multiple physiological pathways beyond target-site mutations.

Biotechnical methods, such as drone brood removal and brood interruption, can reduce mite loads without chemicals and are especially useful in resistant populations (Calderone 2005). The adoption of resistant honey bee strains and breeding for hygienic behavior can also improve colony-level resilience to *Varroa* mites (Boecking and Spivak 1999). Finally, routine monitoring of resistance alleles using molecular diagnostics can inform treatment choices and guide region-specific strategies. Without such adaptive management, resistance is likely to spread, undermining both chemical and biological control efforts (Celikkol and Dogac 2025).

Beekeepers are increasingly cognizant of the detrimental impact of *Varroa* infestations, and the emergence of resistance poses a significant challenge in managing these mites. This issue is particularly urgent for species that have developed resistance to multiple classes of pesticides (Jack and Ellis 2021; Bubnič et al. 2024). The observed regional variationmay reflect both environmental and beekeeping practice differences in these regions. Early detection and management of miticide resistance in *Varroa* destructor are essential, as resistance alleles can rapidly disseminate through populations owing to the haplodiploid reproductive system and high inbreeding rates of the species (Beaurepaire et al. 2017; González-Cabrera et al. 2018). Additionally, it is essential to apply evolutionary principles when utilizing pesticides to minimize the selection pressure for the development of novel resistance mechanisms (Celikkol and Dogac 2025). The rapid evolution of resistance to chemical treatments emphasizes the urgent need for alternative control strategies against these pests. Exacerbating this issue are the detrimental side effects of certain miticides on honey bees(Tihelka 2018), which underscores the necessity of developing more sustainable approaches.

In conclusion, the high prevalence of resistance alleles in Turkish *Varroa* populations calls for a transition from reliance on the same chemical control to a diversified, adaptive management strategy. Regular resistance monitoring and molecular diagnostic tools, such as PCR-RFLP, are essential for maintaining the effectiveness of control measures and preventing the further spread of resistance, thereby supporting sustainable *Varroa* management. The use of different insecticides/organic acids and the implementation of IPM practices can prolong the effectiveness of current treatments and contribute to the sustainability of beekeeping practices in Türkiye. Strengthening Varroa management is essential not only for the health of honeybee colonies but also for safeguarding agricultural productivity and ensuring global food security in the long term.

## Supplementary Information

Below is the link to the electronic supplementary material.

Supplementary Material 1

## Data Availability

The datasets generated during and/or analysed during the current study are available from the corresponding author on reasonable request.

## References

[CR1] Alissandrakis E, Ilias A, Tsagkarakou A (2017) Pyrethroid target site resistance in Greek populations of the honey bee parasite *Varroa destructor* (Acari: Varroidae). J Apic Res 56(5):625–630. 10.1080/00218839.2017.1368822

[CR2] Almecija G, Schimmerling M, Del Cont A, Poirot B, Duquesne V (2022) *Varroa destructor* resistance to tau-fluvalinate: relationship between in vitro phenotypic test and VGSC L925V mutation. Pest Manag Sci 78(12):5097–5105. 10.1002/ps.7126 36103265 10.1002/ps.7126 PMC9826128

[CR3] Anderson DL, Trueman JWH (2000) *Varroa jacobsoni* (Acari: Varroidae) is more than one species. Exp Appl Acarol 24:165–189 11108385 10.1023/a:1006456720416

[CR4] Bahreini R, González-Cabrera J, Hernández-Rodríguez CS, Moreno-Martí S, Muirhead S, Labuschagne RB, Rueppell O (2025) Arising amitraz and pyrethroids resistance mutations in the ectoparasitic *Varroa destructor* mite in Canada. Sci Rep 15(1):1587. 10.1038/s41598-025-85279-6 39794392 10.1038/s41598-025-85279-6 PMC11724071

[CR5] Beaurepaire AL, Krieger KJ, Moritz RF (2017) Seasonal cycle of inbreeding and recombination of the parasitic mite *Varroa destructor* in honeybee colonies and its implications for the selection of acaricide resistance. Infect Genet Evol 50:49–54. 10.1016/j.meegid.2017.02.011 28216419 10.1016/j.meegid.2017.02.011

[CR6] Benito-Murcia M, Martín-Hernández R, Meana A, Botías C, Higes M (2022) Study of pyrethroid resistance mutations in populations of *Varroa destructor* across Spain. Res Vet Sci 152:34–37. 10.1016/j.rvsc.2022.07.021 35917591 10.1016/j.rvsc.2022.07.021

[CR7] Boecking O, Spivak M (1999) Behavioral defenses of honey bees against Varroa jacobsoni Oud. Apidologie 30(2–3):141–158. 10.1051/apido:19990205

[CR8] Bubnič J, Prešern J, Pietropaoli M, Cersini A, Moškrič A, Formato G, Manara V, Smodiš Škerl MI (2024) Integrated Pest Management Strategies to Control *Varroa* Mites and Their Effect on Viral Loads in Honey Bee Colonies. Insects 15(2):115. 10.3390/insects15020115 38392534 10.3390/insects15020115 PMC10889759

[CR9] Calatayud-Vernich P, Calatayud F, Simo E, Pico Y (2018) Pesticide residues in honey bees, pollen and beeswax: assessing beehive exposure. Environ Pollut 241:106–114. 10.1016/j.envpol.2018.05.059 29803024 10.1016/j.envpol.2018.05.062

[CR10] Calderone NW (2005) Evaluation of drone brood removal for management of *Varroa destructor* (Acari: Varroidae) in colonies of Apis mellifera (Hymenoptera: Apidae) in the northeastern United States. J Econ Entomol 98(3):645–650. 10.1603/0022-0493-98.3.645 16022287 10.1603/0022-0493-98.3.645

[CR11] Celikkol E, Dogac E (2025) The status of pyrethroid resistance mutation frequencies in *Varroa destructor* populations in the most important beekeeping areas of Türkiye. Exp Appl Acarol 94:1–14 10.1007/s10493-025-01002-0 PMC11774987 39873781

[CR12] Davies TGE, Field LM, Usherwood PNR, Williamson MS (2007) A comparative study of voltage-gated sodium channels in the Insecta: implications for pyrethroid resistance in Anopheline and other Neopteran species. Insect Mol Biol 16:361–375. 10.1111/j.1365-2583.2007.00733.x 17433068 10.1111/j.1365-2583.2007.00733.x

[CR13] Dong K, Du Y, Rinkevich F, Nomura Y, Xu P, Wang L, Zhorov BS (2014) Molecular biology of insect sodium channels and pyrethroid resistance. Insect Biochem Mol Biol 50:1–17 24704279 10.1016/j.ibmb.2014.03.012 PMC4484874

[CR14] Erdem E, Koç-İnak N, Rüstemoğlu M, İnak E (2024) Geographical distribution of pyrethroid resistance mutations in *Varroa destructor* across Türkiye and a European overview. Exp Appl Acarol 92(3):309–321. 10.1007/s10493-023-00879-z 38401013 10.1007/s10493-023-00879-z PMC11035437

[CR15] González-Cabrera J, Davies TE, Field LM, Kennedy PJ, Williamson MS (2013) An amino acid substitution (L925V) associated with resistance to pyrethroids in *Varroa destructor*. PLoS ONE 8(12):1–6. 10.1371/journal.pone.0082941 10.1371/journal.pone.0082941 PMC3867425 24367572

[CR16] González-Cabrera J, Rodríguez-Vargas S, Davies TE, Field LM, Schmehl D, Ellis JD, Krieger K, Williamson MS (2016) Novel mutations in the voltage-gated sodium channel of pyrethroid-resistant *Varroa destructor* populations from the Southeastern USA. PLoS ONE 11(5):2–8. 10.1371/journal.pone.0155332 10.1371/journal.pone.0155332 PMC4871586 27191597

[CR17] González-Cabrera J, Bumann H, Rodríguez-Vargas S, Kennedy PJ, Krieger K, Altreuther G, Hertel A, Hertlein G, Nauen R, Williamson MS (2018) A single mutation is driving resistance to pyrethroids in European populations of the parasitic mite, *Varroa destructor*. J Pest Sci 91:1137–1144. 10.1007/s10340-018-0968-y

[CR43] Hernández-Rodríguez CS, Marín Ó, Calatayud F, Mahiques MJ, Mompó A, Segura I, Simó E, González-Cabrera J (2021) Large-Scale Monitoring of Resistance to Coumaphos, Amitraz, and Pyrethroids in Varroa destructor. *Insects* 12(1):27. 10.3390/insects12010027 10.3390/insects12010027 PMC7824307 33406622

[CR18] Higes M, Martín-Hernández R, Hernández-Rodríguez CS, González-Cabrera J (2020) Assessing the resistance to acaricides in *Varroa destructor* from several Spanish locations. Parasitol Res 119(11):3595–3601. 10.1007/s00436-020-06879-x 32935162 10.1007/s00436-020-06879-x

[CR19] Hubert J, Nesvorna M, Kamler M, Kopecky J, Tyl J, Titera D, Stara J (2014) Point mutations in the sodium channel gene conferring tau-fluvalinate resistance in *Varroa destructor*. Pest Manag Sci 70(6):889–894. 10.1002/ps.3679 24243563 10.1002/ps.3679

[CR20] Jack CJ, Ellis JD (2021) Integrated Pest Management Control of *Varroa destructor* (Acari: Varroidae), the Most Damaging Pest of (*Apis mellifera* L. (Hymenoptera: Apidae)) Colonies. J Insect Sci 21(5):6. 10.1093/jisesa/ieab058 34536080 10.1093/jisesa/ieab058 PMC8449538

[CR21] Keszthelyi S, Sipos T, Csóka Á, Donkó T (2021) CT-supported analysis of the destructive effects of *Varroa destructor* on the pre-imaginal development of honey bee, *Apis mellifera*. Apidologie 52(1):155–162

[CR22] Koç N, İnak E, Jonckheere W, Van Leeuwen T (2021) Genetic analysis and screening of pyrethroid resistance mutations in *Varroa destructor* populations from Turkey. Exp Appl Acarol 84(2):433–444. 10.1007/s10493-021-00626-2 33983538 10.1007/s10493-021-00626-2

[CR23] Lin Z, Shen S, Wang K, Ji T (2024) Biotic and abioticstresses on honeybee health. Integr Zool 19:442–457. 10.1111/1749-4877.12752 37427560 10.1111/1749-4877.12752

[CR24] Lin Z, Zhang Y, Wang K, Jiang X, Ji T (2025) Genetic Bottlenecks in modern beekeeping: implications for conservation and sustainable pollination. Conserv Lett, 18(6):1–6, e13156. 10.1111/conl.13156

[CR25] Locke B, Thaduri S, Stephan JG, Dahlberg M, de Miranda JR (2021) Adapted tolerance to virus infections in four geographically distinct *Varroa destructor*-resistant honeybee populations. Sci Rep 11:12359. 10.1038/s41598-021-91686-2 34117296 10.1038/s41598-021-91686-2 PMC8196020

[CR27] Marcangeli J, Monetti L, Fernandez N (1992) Malformations produced by *Varroa jacobsoni* on *Apis mellifera* in the province of Buenos Aires, Argentina. Apidologie 23(5):399–402

[CR26] Marsky U, Rognon B, Douablin A, Viry A, Rodríguez Ramos MA, Hammaidi A (2024) Amitraz resistance in French *Varroa* mite populations—more complex than a single-nucleotide polymorphism. Insects 15(6):390 38921105 10.3390/insects15060390 PMC11203491

[CR28] Milani N, Della Vedova G (2002) Decline in the proportion of mites resistant to fluvalinate in a population of *Varroa destructor* not treated with pyrethroids. Apidologie 33(4):417–422. 10.1051/apido:2002028

[CR29] Millán-Leiva A, Marin O, Christmon K, VanEngelsdorp D, González-Cabrera J (2021) Mutations associated with pyrethroid resistance in *Varroa* mites, a parasite of honey bees, are widespread across the USA. Pest Manag Sci 77(7):3241–3249. 10.1002/ps.6366 33728766 10.1002/ps.6366

[CR30] Ogihara MH, Kobayashi E, Morimoto N, Yoshiyama M, Kimura K (2021) Molecular analysis of voltage-gated sodium channels to assess τ-fluvalinate resistance in Japanese populations of *Varroa destructor* (Acari: Varroidae). Appl Entomol Zool 56:277–284. 10.1007/s13355-020-00717-3

[CR31] Panini M, Reguzzi MC, Chiesa O, Cominelli F, Lupi D, Moores G, Mazzoni E (2019) Pyrethroid resistance in Italian populations of the mite *Varroa destructor*: a focus on the Lombardy region. Bull Insectology 72(2):227–232

[CR32] Peck DT, Seeley TD (2019) Mite bombs or robber lures? The roles of drifting and robbing in *Varroa destructor* transmission from collapsing honey bee colonies to their neighbors. PLoS ONE 14(6):e0218392 31226130 10.1371/journal.pone.0218392 PMC6588223

[CR33] Rinkevich FD (2020) Detection of amitraz resistance and reduced treatment efficacy in the Varroa mite, *Varroa destructor*, within commercial beekeeping operations. PLoS ONE 15(1):e0227264 31951619 10.1371/journal.pone.0227264 PMC6968863

[CR34] Robi DT, Temteme S, Aleme M, Bogale A, Getachew A, Mendesil E (2023) Epidemiology, factors influencing prevalence and level of varroosis infestation (*Varroa destructor*) in honeybee (Apis mellifera) colonies in different agroecologies of Southwest Ethiopia. Parasite Epidemiol Control 23:e00325. 10.1016/j.parepi.2023.e00325 37711152 10.1016/j.parepi.2023.e00325 PMC10498395

[CR35] Rosenkranz P, Aumeier P, Ziegelmann B (2010) Biology and control of *Varroa destructor*. J Invertebr Pathol 103:96–119. 10.1016/j.jip.2009.07.016 19909970 10.1016/j.jip.2009.07.016

[CR36] Shen S, Li M, Dong T et al (2025) Gene expression of long non-coding RNAs and messenger RNAs in *Varroa destructor* mites exposed to essential oil of camphor leaves. Exp Appl Acarol 95:45. 10.1007/s10493-025-01071-1 41114887 10.1007/s10493-025-01071-1

[CR37] Sorucu A, Çöl B, Dibek E, Babayeva A (2025) Assessing phenotypic and genotypic resistance to flumethrin in *Varroa destructor* populations in Muğla. Türkiye Insects 16:548 40558978 10.3390/insects16060548 PMC12193500

[CR38] Stara J, Pekar S, Nesvorna M, Erban T, Vinsova H, Kopecky J, Doskocil I, Kamler M, Hubert J (2019) Detection of tau-fluvalinate resistance in the mite *Varroa destructor* based on the comparison of vial test and PCR–RFLP of *kdr* mutation in sodium channel gene. Exp Appl Acarol 77(2):161–171. 10.1007/s10493-019-00353-9 30810851 10.1007/s10493-019-00353-9

[CR41] Tihelka E (2018) Effects of synthetic and organic acaricides on honey bee (*Apis mellifera*) health: a review.Slov Vet Res 55(2):119–40. 10.26873/SVR-422-2017

[CR39] Vlogiannitis S, Jonckheere W, Laget D, de Graaf DC, Vontas J, Van Leeuwen T (2021) Pyrethroid target-site resistance mutations in populations of the honey bee parasite *Varroa destructor* (Acari: Varroidae) from Flanders, Belgium. Exp Appl Acarol 85(2):205–221. 10.1007/s10493-021-00665-9 34676469 10.1007/s10493-021-00665-9

[CR40] Wang R, Huang ZY, Dong KE (2003) Molecular characterization of an arachnid sodium channel gene from the varroa mite (*Varroa destructor*). Insect Biochem Mol Biol 33(7):733–739 12826100 10.1016/s0965-1748(03)00068-7

[CR42] Yarsan E, Yilmaz F, Sevin S, Akdeniz G, Celebi B, Ozturk SH, Ayikol SN, Karatas U, Ese H, Fidan N, Agacdiken B, Babur C, Buldag M, Pehlivan S (2024) Investigation of resistance against to flumethrin using against *Varroa destructor* in Türkiye. Vet Res Commun 48:1683–1696. 10.1007/s11259-024-10351-x 38509424 10.1007/s11259-024-10351-x PMC11147911

