# Supplementary material for: Widespread pyrethroid resistance in Varroa destructor in Türkiye: a molecular warning
Source: Exp Appl Acarol. 2026 Apr 1;96(3):39. doi: 10.1007/s10493-026-01132-z (PMC13043529; doi:10.1007/s10493-026-01132-z)
Supplement: Supplementary file 1 — Supplementary Material 1 [file 10493_2026_1132_MOESM1_ESM.docx]

**Widespread pyrethroid resistance in *Varroa destructor* in Türkiye: A Molecular Warning**

Experimental and Applied Acarology

Sezer YALCIN^1^, Taylan DOGAROGLU^2^, Evin GUNENC^1^, Ersin DOGAC^3^

^1^ Institute of Science, Department of Molecular Biology and Genetics, Mugla Sıtkı Koçman University, Mugla, Türkiye

^2^ Ula Ali Koçman Vocational School, Department of Plant and Animal Production, Beekeeping Program, Muğla Sıtkı Koçman University, Mugla, Türkiye

^3^ Faculty of Science, Department of Molecular Biology and Genetics, Mugla Sıtkı Koçman University, Mugla, Türkiye

Corresponding author. E-mail: [ersindogac@mu.edu.tr](mailto:ersindogac@mu.edu.tr)

**Online Resource 1** Province-specific pyrethroid resistance status of *Varroa* mites.

| Provinces | Sample size | Number of homozygous resistant individuals (RR) | Number of heterozygous resistant individuals (RS) | Number of homozygous susceptible individuals (SS) |
| --- | --- | --- | --- | --- |
| Yalova | 80 | 32 | 36 | 12 |
| Balıkesir | 80 | 70 | 10 | 0 |
| Tekirdağ | 80 | 68 | 12 | 0 |
| Ankara | 80 | 48 | 28 | 4 |
| Konya | 80 | 78 | 2 | 0 |
| Kayseri | 80 | 66 | 14 | 0 |
| Ardahan | 80 | 74 | 2 | 4 |
| Van | 80 | 74 | 6 | 0 |
| Bingöl | 80 | 76 | 4 | 0 |
| Şırnak | 80 | 76 | 2 | 2 |
| Total | **800** | **662** | **116** | **22** |
